# Immunofluorescence laser micro-dissection of specific nephron segments in the mouse kidney allows targeted downstream proteomic analysis

**DOI:** 10.14814/phy2.12306

**Published:** 2015-02-13

**Authors:** Radmila Micanovic, Shehnaz Khan, Tarek M El-Achkar

**Affiliations:** Division of Nephrology, Indiana University and the Roudebush Indianapolis VA Medical CenterIndianapolis, Indiana

**Keywords:** 2D-DIGE, laser micro-dissection, mass spectrometry, proteomics

## Abstract

Laser micro-dissection (LMD) is a very useful tool that allows the isolation of finite areas from tissue specimens for downstream analysis of RNA and protein. Although LMD has been adapted for use in kidney tissue, the use of this powerful tool has been limited by the diminished ability to identify specific tubular segments in the kidney. In this study, we describe a major improvement in the methodology to isolate specific cells in the mouse kidney using immunofluorescence LMD (IF-LMD). Using IF-LMD, we can reproducibly isolate not only glomeruli, but also S1–S2 proximal segments, S3 tubules, and thick ascending limbs. We also demonstrate the utility of a novel rapid immunofluorescence staining technique, and provide downstream applications for IF-LMD such as real-time PCR and cutting-edge proteomic studies. This technical breakthrough may become an invaluable tool for understanding cellular and molecular events in the heterogeneous kidney milieu.

## Introduction

Laser micro-dissection (LMD) is a tool developed in the last two decades that allows direct visualization of tissue sections and capture/isolation of a finite number of cells (Espina et al. 2006). These cells can be used for downstream applications such as RNA or protein extraction and subsequent analyses. In the kidney, LMD was first described by Star and colleagues, who were successful in isolating various tubular segments using rapid immunohistochemical techniques (Kohda et al. 2000). Subsequently, the same group adapted immunofluorescence to LMD (IF-LMD), and were able to isolate thick ascending limbs (TAL) using a specific marker (Murakami et al. 2000). Since those seminal studies, the use of LMD in kidney tissue has been mostly focused on isolation of glomeruli, which are easily recognizable (Pietrzyk et al. 2004; Woroniecki and Bottinger 2009; Wang et al. 2010; Sethi et al. 2012). It is not fully clear why the use of LMD to isolate various tubular segments has not gained traction, despite important advances (Noppert et al. 2011; Wilkinson et al. 2014). The cause could be partly due to the difficulty in recognizing tubular segments, especially to nonexpert users. With the availability of newer, user-friendly platforms for LMD, and the access to novel reagents for tissue handling and processing, the ability to reliably dissect specific tubular segments for RNA and protein isolation is now reachable. In this study, we describe a novel methodology to stain kidney tissue for IF-LMD, and demonstrate downstream applications for LMD such as RNA extraction and state-of-the-art protein analysis.

## Materials and Methods

### Mice

Animal experiments and protocols were approved by the Indianapolis VA Animal Care and Use Committee. Age-matched 12-week-old Tamm-Horsfall protein (THP) knockout mice (129/SvEv THP−/−) and wild-type background strain were used as previously (El-Achkar et al. 2011, 2013).

### Immunofluorescence confocal microscopy

Immunofluorescence staining for Na^+^-K^+^-ATPase (Santa Cruz Dallas, TX, Sc-28800), DAPI and FITC-phalloidin (Molecular probes part of Life Technologies, Grand Island, NY) was done on 50 *μ*m vibratome sections of kidneys fixed with 4% paraformaldehyde as described previously (El-Achkar et al. 2013).

### Immunofluorescence LMD (IF-LMD)

Kidneys were extracted after euthanasia. Sections from each kidney were snap frozen in OCT on dry ice and kept at −80°C until use. They were subsequently cut using a microtome at 10 *μ*m sections on Leica PPS membrane slides (Cat #11505268). For dissection of glomeruli and proximal segments, we used the following sequence of staining: (1) 100% EtOH for 30 sec × 2, (2) 95% EtOH for 20 sec × 2, (3) 75% EtOH for 20 sec × 2, (4) 50% EtOH for 20 sec × 2, (5) Water 1, 30 sec × 2, (6) Water 2, 30 sec × 2, (7) staining with FITC-phalloidin (1:20)+ DAPI (molecular probes, 1:300) in PBS+2% BSA for 3–5 min (we use 75 *μ*L to cover one section, and we typically have two sections cut on one membrane slide), (8) Phosphate buffer wash for 30 sec × 3, (9) air dry for 5 min.

For staining of TAL we used the sequence 1–6 as above, then (7) Anti-Na^+^-K^+^-ATPase (1:5)+ FITC-phalloidin (1:20)+ DAPI (1:300) in PBS+2% BSA for 4–5 min, (8) Phosphate buffer wash for 30 sec × 2, (9) secondary-conjugated Ab (Alexa 555 Donkey anti-Rabbit from molecular probes) × 4 min, (10) Phosphate buffer wash for 30 sec × 1, (11) air dry for 5 min.

Sections were immediately taken to a Leica LMD6000 laser micro-dissection microscope. Dissection was performed at 40× magnification under fluorescence. 200–250 segments are dissected in each 90 min session (average dissected area varies on type of tubules, for S3 segments range: 350,000–500,000 *μ*m^2^).

### RNA extraction and real-time PCR

RNA was extracted using Pico-pure RNA kit (Life *Technologies* # 12204-01). An additional concentration step was performed using standard isopropanol precipitation, before reverse transcription and real-time PCR. We typically pool and concentrate RNA extracted from 1000 tubules over the course of 4–6 LMD sessions. We test the integrity of the RNA by running real-time -PCR for GAPDH, similar to what is reported (Noppert et al. 2011), and typically target a CT of 26–29 for GAPDH in LMD samples. The purity of the specific tubular segments isolated was verified by the absence of markers for other segments, as we showed recently (Micanovic et al. 2015). RNA from total kidney (extracted also from the stained sections that were used for LMD) was used as positive control for all markers. We used the following Taqman gene expression assays all from Applied Biosystems: SGLT2 (Mm00453831_m1), NKCC2 (Mm00441424_m1), rBAT (Mm00486218_m1), Podocin (Mm01292252-m1) and GAPDH (Mm99999915_g1) as endogenous control**.** Samples are run in triplicates. A t-test was used to compare the difference between two groups, at a significance level <0.05.

### Protein extraction, two-dimensional differential gel electrophoresis (2D-DIGE) and Mass spectrometry

Tubules dissected using IF-LMD were treated with protein extraction cocktail: (all from Sigma, St Louis, MO) 0.5% Nonidet P-40, 1% TritonX, 300 mmol/L NaCl, 20 nmTris pH 7.5, 2 mmol/L EDTA, 2 mmol/L EGTA, Leupeptin (1 *μ*g/mL), Antipain (2 *μ*g/mL), Benzamidine (10 *μ*g/mL), Chymostatin (1 *μ*g/mL), Pepstatin (1 *μ*g/mL), Pefabloc SC (24 *μ*g/mL),40 mmol/L Na Fluoride, 1 mmol/L Na Molybdate, 5 mol/L Na Vanadate. Protein measurements were done using a Bradford assay. 2D-DIGE and all subsequent Mass spectrometry analysis were performed at Applied Biomics (Hayward, CA). An initial analytical gel was done by using 50 *μ*g of protein extract from each S3 sample (THP−/− and THP+/+). Each sample was labeled with Cy2 or Cy3, mixed and separated on a 2D gel. In-gel data analyses for protein spots and comparison of the integrated volumetric ratios were done using the DeCyder software, and a fold change >1.5 was used as a cutoff. Protein Spots of interest were digested and extracted from a preparative gel and identified by mass spectrometry (MALDI-TOF and MALDI-TOF/TOF) on the basis of peptide fingerprint mass mapping and peptide fragmentation mapping. The MASCOT search engine was used to identify proteins from primary sequence databases.

## Results

### Laser micro-dissection of nephron segments in the mouse kidney and RNA analysis with real-time PCR

#### A-Glomeruli, S1–S2 and S3 segments

**Figure 1:**
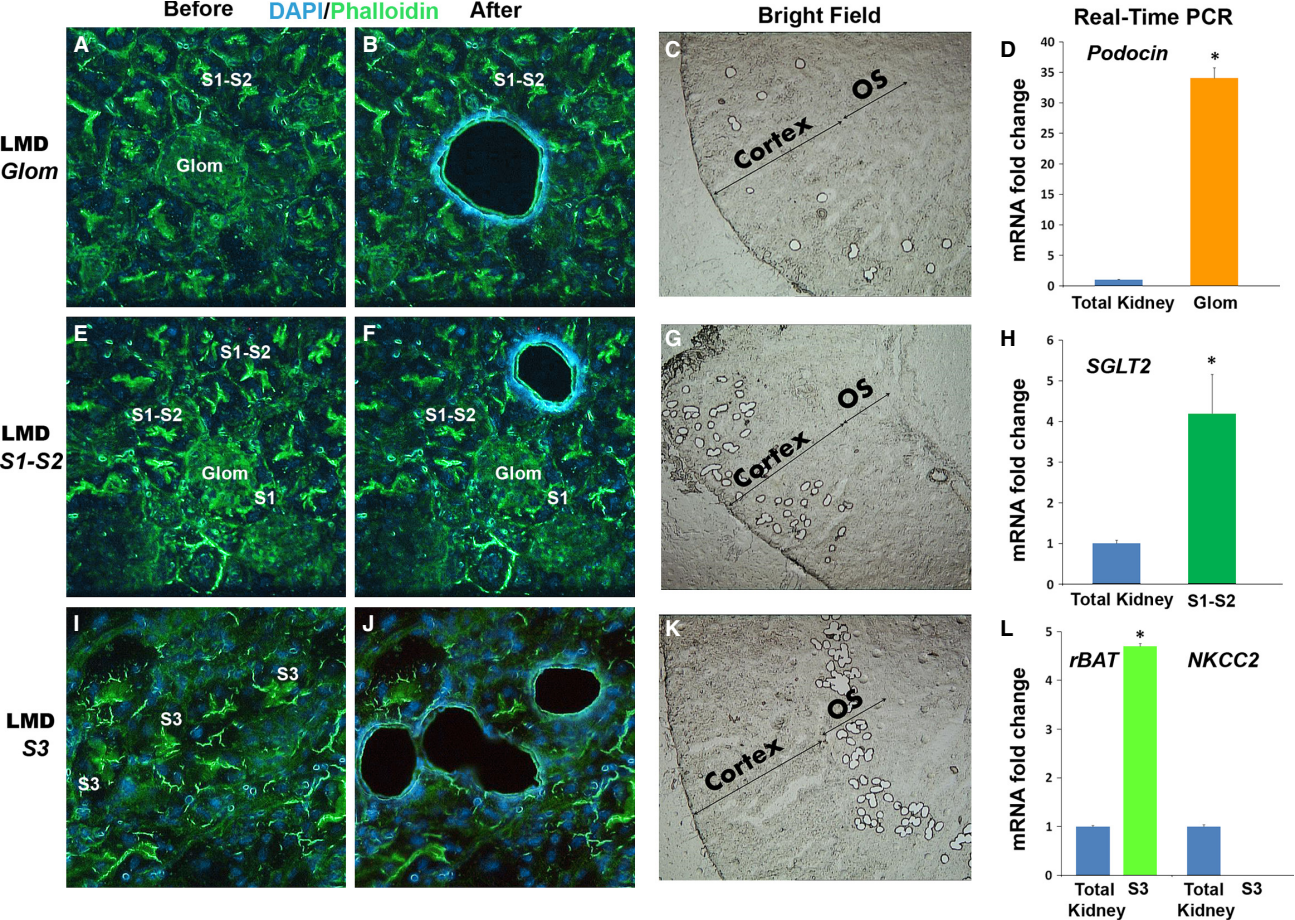
Immunofluorescence laser micro-dissection (IF-LMD) of glomeruli, S1–S2, and S3 proximal tubules. Representative images of IF-LMD of glomeruli, S1–S2 and S3 segments under 40× objective magnification are shown in panels A–B, E–F, and I–J, respectively. Staining was performed using DAPI/Phalloidin. C, G, and K are low magnification (5× objective) bright field microscopy images of kidney sections after dissection of glomeruli, S1–S2 and S3 segments, respectively. Real-time PCR for specific markers (Podocin for glomeruli, SGLT2 for S1–S2, rBAT for S3 segments) was performed on each dissected segment, respectively, as shown in D, H, L, and compared to total kidney used as reference. As expected, each nephron segment had a high level of its corresponding marker as compared to total kidney (*P < 0.05). The purity of RNA sample was demonstrated for S3 segments by the absence of NKCC2, a marker for neighboring thick ascending limb cells in the outer stripe of the outer medulla.

Dissection of glomeruli and proximal tubular segments (S1–S2 and S3) was performed after staining with DAPI (nuclear stain) and phalloidin (actin-binding stain). As shown in Figure1, glomeruli can be easily identified (see also Video S1), and are typically surrounded by S1 and S2 proximal segments, recognized by the typical brush border intense staining with phalloidin. S3 segments, also identified by the intense brush border stain, can be dissected in the outer medulla (OM) (see also Video S2). RNA extracted from each tubular segment was then analyzed using real-time PCR and we validated the expression of segment specific markers for each isolated cell type (Fig.1D, H, and L).

#### B- Thick ascending limbs (TAL) of the loop of Henle

**Figure 2:**
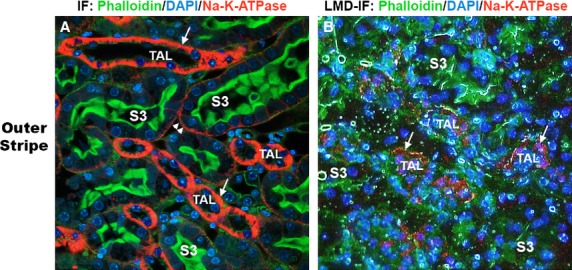
Rapid identification of TAL using Immunostaining NA^+^-K^+^-ATPase. Panel A shows a high-magnification (40× objective) immunofluorescence image of a vibratome-cut, outer medulla kidney section, stained for Na^+^-K^+^-ATPase (red), proximal tubular brush border with phalloidin (green), and DAPI for nuclei (blue). Note the thick red staining on the basolateral domain of TAL segments (arrows) as compared to S3 proximal segments. In B, a rapid sequence immuno-fluorescence staining on tissue prepared for LMD reproduced a similar staining pattern, allowing quick and accurate identification of TAL segments.

**Figure 3:**
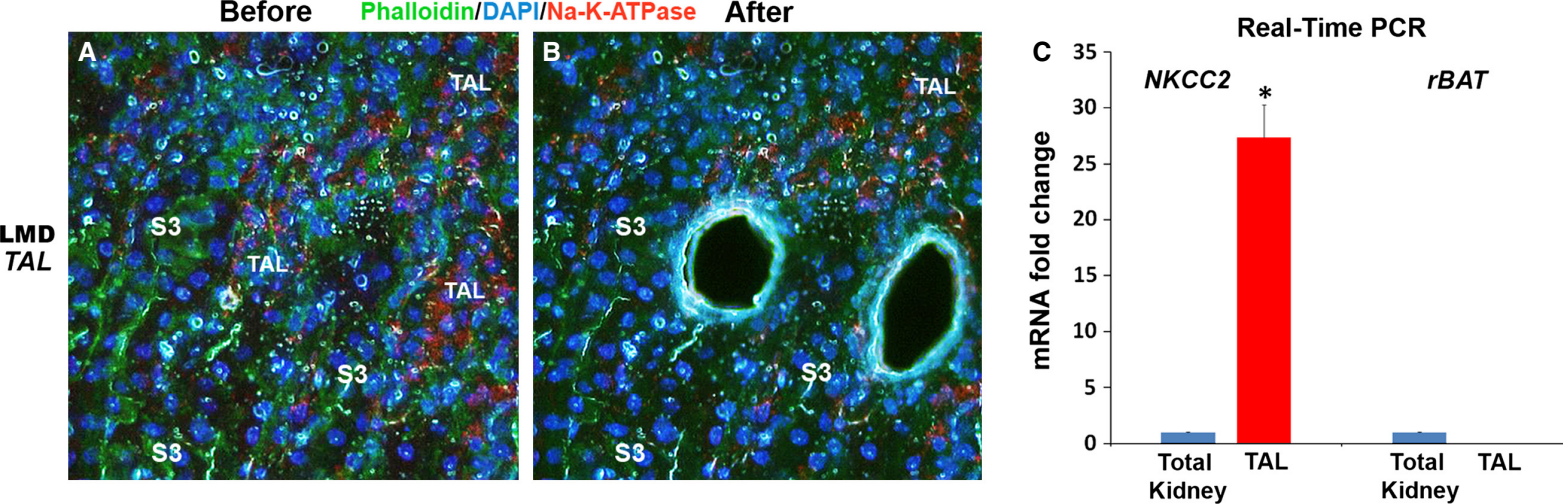
IF-LMF of TAL segments in the outer medulla. Representative images of IF-LMD of TAL segments from the outer medulla in a kidney section stained with Na^+^-K^+^-ATPase/DAPI/Phalloidin is depicted in A and B. Real-time PCR on RNA extracted from TAL cells demonstrates the increased expression of NKCC2 (specific marker) in TAL segments as compared to total kidney (*P < 0.05). The purity of the isolated RNA from TAL was demonstrated by the absence of rBAT (marker for neighboring S3 cells in outer stripe).

To identify TAL segments, we made use of the fact that these segments express very densely Na^+^-K^+^-ATPase on the basolateral domain as shown previously (El-Achkar et al. 2013) and in Figure2A. Therefore, we devised a rapid LMD-Immunofluoresence stain, which easily can identify TAL segments, especially when combined with proximal tubule staining, as shown in Figure2B. Dissection of thick ascending limbs after staining with Na^+^-K^+^-ATPase/DAPI/Phalloidin can then be performed in any area of the kidney. We typically dissect TAL in the OM (Fig.3A). RNA extracted from TAL cells show very high expression of NKCC2, which is uniquely expressed by these cells (Fig.3B).

### Proteomic analysis of S3 segments isolated by IF-LMD using two-dimensional differential gel electrophoresis (2D-DIGE) and Mass Spectrometry

**Figure 4:**
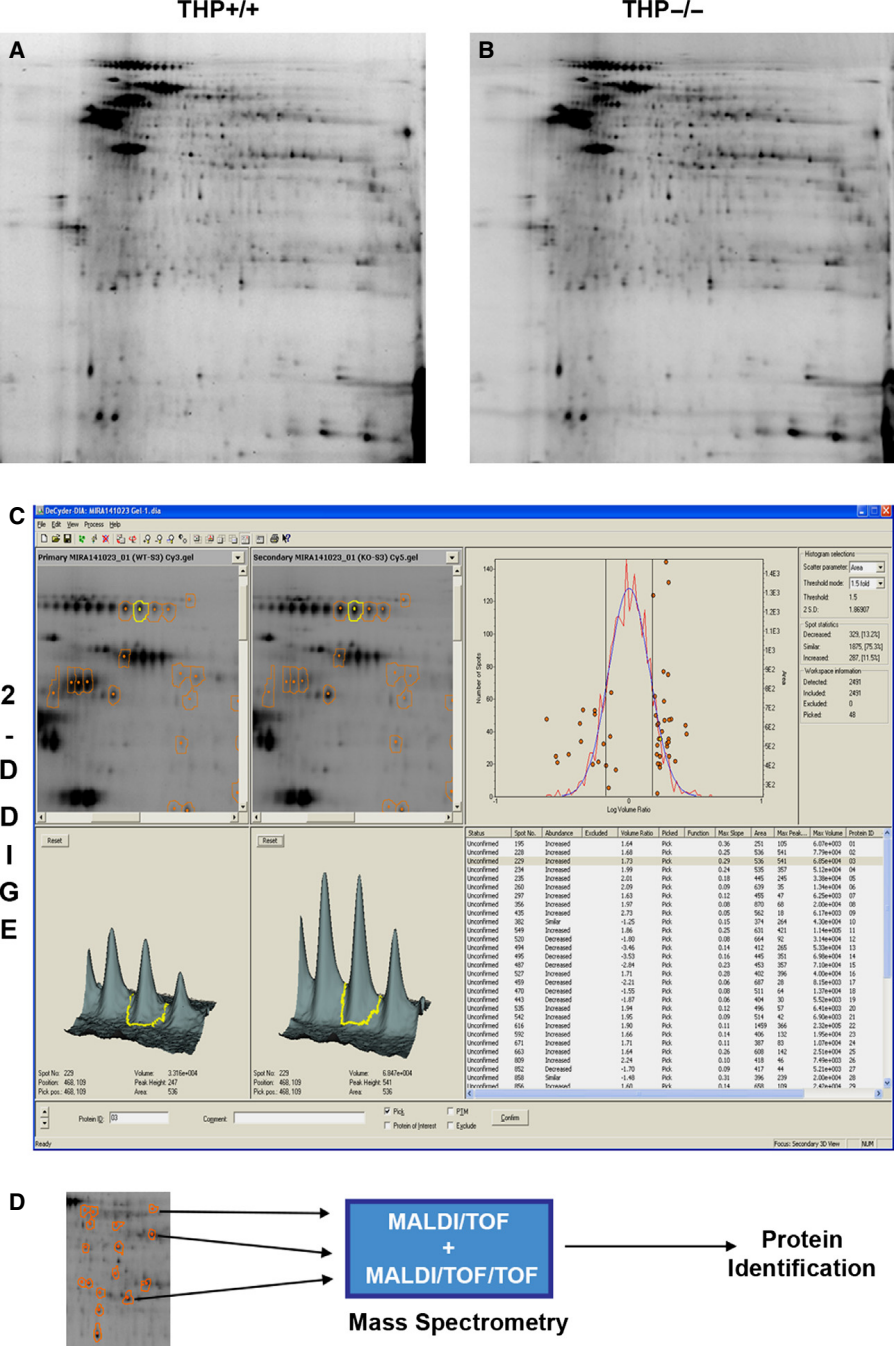
Proteomic analysis of S3 segments from THP−/− and THP+/+ kidneys. Protein extracted from S3 segments isolated by IF-LMD from THP+/+ and THP−/− mice (pooled from *n* = 5 for each group) underwent two-dimensional differential gel electrophoresis (A and B, respectively). In-gel analysis (C) was done using the DeCyder software to analyze protein spots for comparative integrated volumetric measurements (an example for the spot in yellow is shown comparing the volumetric ratio between THP+/+ and THP−/−). A ratio of >1.5 fold change between spots in THP−/− versus THP+/+ was used to identify differentially expressed proteins (Right panel in C). Among those, 48 spots were picked (marked in orange circles, and shown in the distribution graph on top Right), based on the high likelihood of successful identification by mass spectrometry (spot resolution and protein abundance). The majority of these spots were subsequently identified according to the algorithm depicted in D.

**Figure 5:**
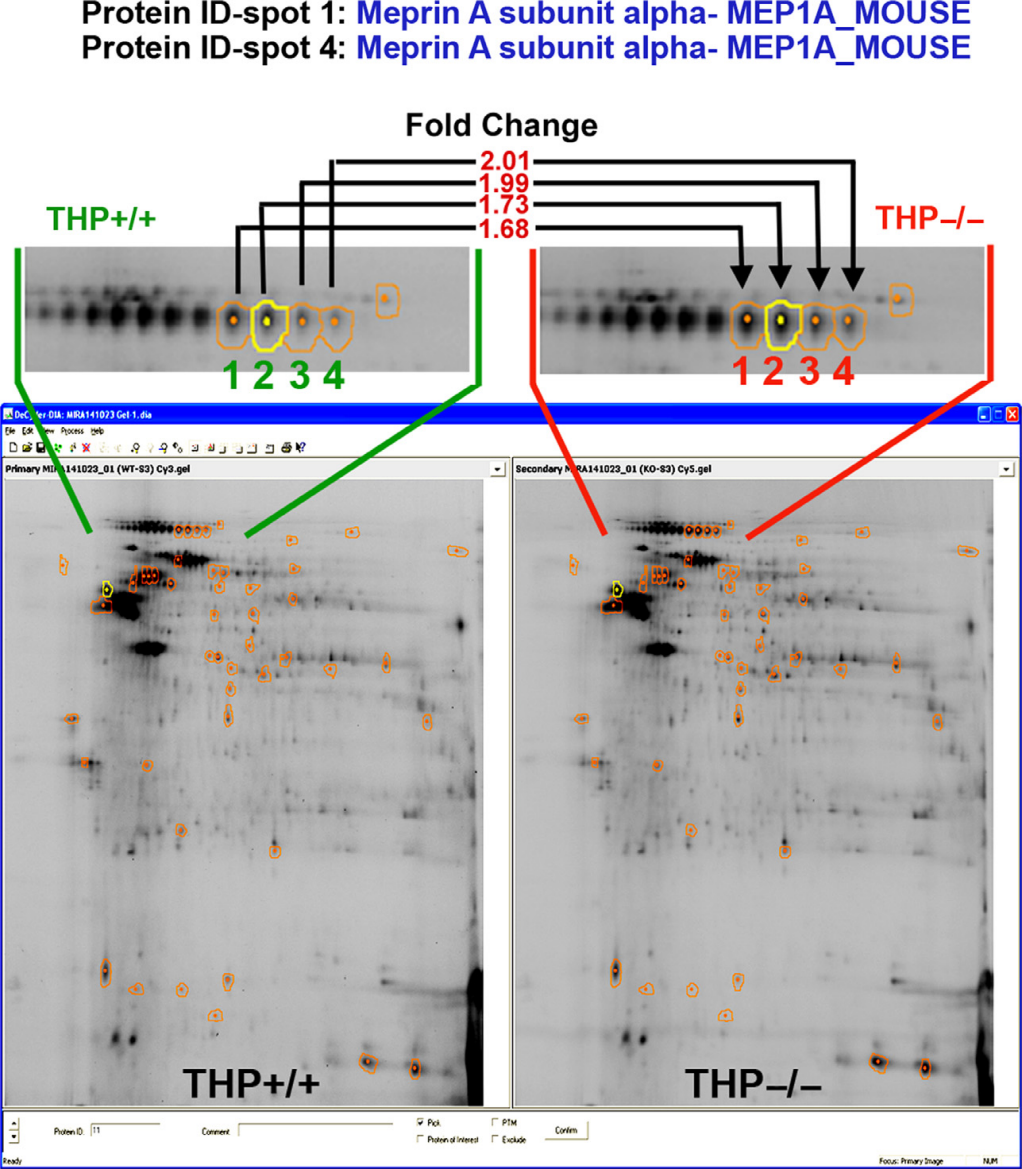
Identification of Meprin A as an example of a protein significantly increased and modified in S3 segments of THP−/− kidneys. Full image of the two-dimensional differential gel electrophoresis and in-gel analysis as described in Figure4 is shown again. Enlarged areas of interest for spots 1–4 from THP+/+ and THP−/− images are outlined with green and red, respectively. The corresponding fold changes between the two strains are also shown. Using mass spectrometry, Meprin A was identified as a significantly increased protein (spot 1 and 4 were identified as Meprin A with 100% confidence, suggesting that spots 2 and 3 are also Meprin A) in THP−/− compared to THP+/+ S3 segments. The lateral smearing of Meprin A on 2D gel suggests posttranslational modifications. Few of these changes were identified as oxidation or carbamidomethylation (not shown).

S3 segments isolated by IF-LMD were processed for proteomic studies. Figure4 shows a two-dimensional differential gel electrophoresis (2D-DIGE) performed on S3 segments isolated from wild-type (THP+/+) and Tamm-Horsfall Protein knockout (THP−/−) mice using IF-LMD. In-gel data analyses were performed using DeCyder software. A total of 2491 spots were detected. Using a fold change of ≥1.5 was as cutoff for differentially expressed proteins in S3 segments from THP−/− versus THP+/+ mice, 287 spots (11.5%) were increased, whereas 329 spots (13.2%) were decreased. From these differentially expressed spots, 48 spots were picked by DeCyder based on high likelihood of successful identification by mass spectrometry (well separated spots, good amount of protein by volume computation). Few spots of interest with important changes in protein expression (just below the 1.5 cut off) were also included based on protein abundance by volume computation. Spot identification was done using mass spectrometry (MALDI/TOF and MALDI/TOF/TOF), and most of the picked spots were identified (Data not shown, will be described in detail in a subsequent report). Figure5 shows an example, where we identified that Meprin A was significantly increased and modified in S3 segments of THP−/− compared to THP+/+ kidneys.

## Discussion

In the current study, we describe major advancements in laser micro-dissection of specific cells within the kidney using a novel immunofluorescence staining method. We validate specific stains for proximal segments and TAL cells. We also show novel downstream applications of LMD by performing proteomic analysis. We believe that this technical innovation will make LMD more adaptable to kidney research.

The application of IF-LMD to the kidney has been pioneered by Star and colleagues, who successfully isolated tubular thick ascending limbs using immunofluorescence (Kohda et al. 2000; Murakami et al. 2000). LMD has been since used to isolate glomeruli predominantly (Pietrzyk et al. 2004; Woroniecki and Bottinger 2009; Wang et al. 2010), and less so proximal tubules (Noppert et al. 2011; Wilkinson et al. 2014). Sethi and colleagues have innovatively adapted LMD to be used in human kidney biopsies for identification of typing of renal amyloid deposits in the glomeruli and interstitium (Sethi et al. 2012). We recently used LMD with a histochemical stain to show the specific site of expression of inflammatory cytokines within the kidney, such as IL-23 in S3 segments (Micanovic et al. 2015). To our knowledge, the use of immunofluorescence with LMD has not been widely adapted to isolate specific nephron segments. This could be partially related to the difficulty in identifying tubular segments. In the current work, we provide a reliable immunofluorescence protocol that could be used to identify specific nephron segments based on the expression of specific markers. We anticipate that this protocol can be used to stain for other markers and protein of interest, which would enable the isolation of specifically labeled cells in the kidneys.

The downstream application of IF-LMD in proteomic analysis using 2D-DIGE followed by mass spectrometry to identify differentially expressed proteins in specific nephron segments is very novel. We show here an example how this tool can be used to uncover differentially expressed protein in S3 segments from THP−/− compared to THP+/+ kidneys. To our knowledge, this is the first instance of using proteins extracted from specific tubular segments for advanced proteomic analysis. We anticipate that this methodology will be useful to unravel signaling events in specific tubules during various experimental conditions. For example, the identification of Meprin A as a differentially increased protein in S3 segments of THP-deficient mice is of great interest. Meprin A is a metalloendoprotease localized in the proximal tubules in the kidney (Kaushal et al. 2013). In addition, Merpin is known to promote inflammation during AKI (Kaushal et al. 2013). Therefore, the activation of Meprin A in S3 segments of THP−/− mice could partially explain the proinflammatory phenotype exhibited by these cells in THP deficiency (El-Achkar et al. 2013; Micanovic et al. 2015), and warrants further investigation.

In conclusion, the current technical study significantly optimized the use of IF-LMD to isolate specific tubular segments within the kidney and perform cutting-edge analysis on the extracted RNA and protein. This technical breakthrough will become essential for understanding cellular and molecular events in the heterogeneous kidney milieu.

## Supporting Information

**Video S1.** Immunofluorescence laser micro-dissection (IF-LMD) of a Glomerulus.

**Video S2.** Immunofluorescence laser micro-dissection (IF-LMD) of an S3 segment in the outer medulla.
