# Supplementary material for: Immunofluorescence laser micro-dissection of specific nephron segments in the mouse kidney allows targeted downstream proteomic analysis
Source: Physiol Rep. 2015 Feb 13;3(2):e12306. doi: 10.14814/phy2.12306 (PMC4393212; doi:10.14814/phy2.12306)
Supplement: Supplementary file 3 [file phy20003-e12306-sd3.docx]

**Legend for supplemental material**

**Supplemental Video 1: Immuno-fluorescence Laser Micro-dissection (IF-LMD) of a Glomerulus**

A glomerulus is dissected under high power magnification after staining with Phalloidin (green) and DAPI (blue). Note the proximal tubules (S1 and S2) around the glomerulus with intense brush border staining with Phalloidin

**Supplemental Video 2: Immuno-fluorescence Laser Micro-dissection (IF-LMD) of an S3 segment in the outer medulla**

A cross-sectional S3 tubule is dissected under high power magnification after staining with Phalloidin (green) and DAPI (blue). Note the intense staining of the brush border with Phalloidin.
